# Nutritional value, micronutrient and antioxidant capacity of some green leafy vegetables commonly used by southern coastal people of Bangladesh

**DOI:** 10.1016/j.heliyon.2019.e02768

**Published:** 2019-11-19

**Authors:** S.M. Neamul Kabir Zihad, Yashu Gupt, Shaikh J. Uddin, Muhammad Torequl Islam, Md. Rabiul Alam, Shahin Aziz, Mahmood Hossain, Jamil A. Shilpi, Lutfun Nahar, Satyajit D. Sarker

**Affiliations:** a Pharmacy Discipline, Khulna University, Khulna, 9208, Bangladesh; b Department for Management of Science and Technology Development, Ton Duc Thang University, Ho Chi Minh City, 700000, Viet Nam; c Faculty of Pharmacy, Ton Duc Thang University, Ho Chi Minh City, 700000, Viet Nam; d Forestry and Wood Technology Discipline, Khulna University, Khulna, 9208, Bangladesh; e Bangladesh Council of Scientific and Industrial Research, Dhaka, Bangladesh; f Medicinal Chemistry and Natural Products Research Group, School of Pharmacy and Biomolecular Sciences, Liverpool John Moores University, Liverpool, United Kingdom

**Keywords:** Natural product chemistry, Food analysis, Food composition, Food chemistry, Nutrition, Nutrient availability, Coastal vegetables, Nutritional and antioxidant potential, Micronutrient, Free radical

## Abstract

Southern coastal people of Bangladesh are highly vulnerable to food insecurity and malnutrition due to coastal flooding, deforestation and increased soil salinity. A number of green leafy vegetables are found in the southern coastal belt being traditionally eaten as daily basis by local people. But they are unaware of nutritional and medicinal use of these vegetables. To contribute to their wider utilization, five common vegetables namely *Hibiscus sabdariffa, Trianthema portulacastrum*, *Diplazium esculentum*, *Heliotropium indicum L*. and *Hygrophila auriculata* were selected for analysis of nutritional proximate, micronutrients and antioxidant potential. Nutritional properties were analyzed in terms of moisture, pH, protein, lipid, ash, fibre, minerals and carbohydrate. Total flavonoid, tannin and antioxidant capacity were evaluated using established protocols. The results demonstrated that collected plants are rich in carbohydrate, fibre, proteins, moisture and ash content but low in lipid content. The mineral elements were high with remarkable amount of Na (19.9–21.5 mg/gm), K (7.9–13.5 mg/gm) and P (1.0–1.8 mg/gm). All the samples were found to have considerable amount of flavonoid (90.6–144.5 mg QE/gm) and tannin content (26.8–57.2 mg GAE/gm). The IC_50_ value of DPPH and superoxide radical scavenging was the lowest for *H. indicum* (37.1 and 83.4 μg/ml, respectively) whereas *T. portulacastrum* possessed high reducing power (IC_50_ 53.7 μg/ml). Among the five investigated species, *T. portulacastrum* and *H. indicum* were found to have good nutritional and antioxidant properties, thus can be promoted as a significant source of nutritional and antioxidant food supplements.

## 1 Introduction

Food security is one of the global problems and exists for decades. Lack of minerals, vitamins and other nutritional elements can cause depletion of respective antioxidant enzymes level which led to oxidative stress [1]. Malnutritional people are more vulnerable to infections and many other diseases through continuous oxidative stress on cellular systems. Oxidative stress often associated with the generation of reactive oxygen species (ROS) including free radicals and strongly implicated in the pathophysiology of diseases, such as cancer, rheumatoid arthritis, alzheimer's disease, parkinson's disease, neurodegeneration, aging, cirrhosis, arteriosclerosis etc [2]. Antioxidants neutralize free radicals by donating electrons and helping to prevent cell and tissue damage [3]. Antioxidant supplements or antioxidant-containing foods play an important role in protection of oxidative damage when endogenous mechanism of antioxidant protection becomes unbalanced. Phenolic compounds, protein hydrolyzates and some amino acids, present in different food, were found to have antioxidant properties because of their free radical scavenging ability [4]. Foods rich in natural polyphenol can prevent hyperlipidaemia. However, the most commonly used synthetic antioxidants at the present time (such as butylated hydroxyanisole (BHA), butylhydroxytoluene (BHT), propyl gallate and tert-butylhydroxyquinone) have restricted use in foods as they are suspected to be carcinogenic and to cause liver damage. Therefore, around the world there is currently great interest in studying the dietary supplements (in foods) containing natural antioxidants that can protect the human body from free radicals and retard the progress of many chronic diseases [3, 5].

Bangladesh is the world's eight-most populous country (over 160 million people). Because of huge demand for food, the nation has always been struggling against poverty and starvation [6, 7]. Malnutrition in Bangladesh is among the highest in the world. More than 54% of pre-school children and 50% of women suffer from chronic nutritional deficiency. Due to rapid population growth and limited cultivable land in Bangladesh, we are struggling to meet the high demand of food. Recently chronic life threatening health problems such as diabetes, cardiovascular disease, cancer and obesity are becoming very common in Bangladesh which are creating tremendous pressure on health care delivery system [8]. Therefore, it is very urgent need to find out diversify food sources in terms of both land and environmental sustainability to satisfy the nutritional requirements of the people in Bangladesh [8]. Because of their high nutritional and medicinal value, use of vegetables can play an important role in improving the nutritional status of the population and can prevent a number of common diseases.

Bangladesh ranked the most vulnerable country for potential negative impact on agricultural production and food security challenges because of climate changes, accelerated sea level rise associated with flood, cyclone and coastal strom [9]. In coastal belt especially in Khulna, one of the main food security challenges is soil salinity (due to salt intrusion) and lack of farming land due to shrimp farming [10]. Coastal plants are rich source of nutrients and energy. Many leafy vegetable are mainly seasonal and some are native grown all-round the year especially in the coastal belt areas of Bangladesh. These leafy vegetables are important to the life of the dwellers of the area. These leafy vegetables are one of the main food items of coastal people that they intake daily basis as cooked food. However, there is no scientific exploration of these resources to increase awareness of its beneficiary effects in terms of nutritional and medicinal importance of these leafy vegetables although they constitute a large proportion of the daily diet of the coastal dweller of the country. In order to find dietary source with full of antioxidant and nutrient that can prevent oxidative damage induced different diseases, these green plants can play an important role in improving the nutritional status of the poor coastal people and prevent a number of common diseases in Bangladesh.

In this study, five (05) green leafy vegetable, belongs to five different families, commonly used by the southern coastal region people of Bangladesh were collected from coastal region of Dumoria, Khulna, Bangladesh **(**Table 1). These vegetables have a long history of ethnomedicinal use and many of their pharmacological properties are scientifically demonstrated (Table 2). The overall objective of this was to explore the nutritional and antioxidant potential of these green leafy vegetables with the goal to increase awareness of the beneficial effects of these leafy vegetables among the coastal peoples of Bangladesh.

**Table 1:** List of collected plants commonly used by southern coastal people of Bangladesh.

S.N.	Botanical Name	Common Name	Family	Voucher no.
1	*Diplazium esculentum*	Dheki	Athyriaceae	DACB 43181
2	*Heliotropium indicum*	Hatisur	Boraginaceae	DACB 43134
3	*Hibiscus sabdariffa*	Taksak	Malvaceae	DACB 43135
4	*Hygrophila auriculata*	Kulekaanta	Acanthaceae	DACB 43136
5	*Trianthema portulacastrum*	Gadabani	Aizoaceae	DACB 44926

**Table 2:** Ethnobotanical use and reported pharmacological activity of the collected leafy vegetables.

Name of vegetable	Family	Local name	Ethnobotanical use	Reported Pharmacological activity	Ref.
*Diplazium esculentum*	Athyriaceae	Dheki	• Cough • Asthma • Phthisis • Fever • Dyspepsia • Stomachache • Diarrhea • Insect and pest repellant • Haemoptysis • Constipation	• Antioxidant • Cytotoxic • Antimicrobial • CNS stimulant • Anthelmintic	[64, 65, 66, 67]
*Heliotropium indicum*	Boraginaceae	Hatisur	• Ulcers • Sores • Wounds • Gum boils • Stings of insects • Rheumatism • Gonorrhea • Putrefaction • Pyoderma • Ringworm infection • Diuretic • Intractable fever • Sore throat • Eye lotion • Whooping cough in children	• Antitumor • Antimicrobial • Anti-inflammatory • Wound healing • Anti-proliferative • Anti-tuberculosis • Gastro protective • Immuno stimulant • Antioxidant • Antihyperglycemic • Antihelmintic	[68, 69, 70, 71, 72, 73, 74, 75, 76, 77, 78, 79, 80, 81, 82]
*Hibiscus sabdariffa*	Malvaceae	Taksak	• Diuretic • Gastrointestinal disorders • Liver diseases • Fever • Hypercholesterolemia • Hypertension • Sore throat • Cough • Stomachic • Emollient	•hepatoprotective • antioxidant • anti-obesity • anticholesterol • anticancer • inhibition of the contractility of rat bladder and uterus • antibacterial • antihypertensive • Anti-anaemic • Anti-diabetic • Diuretic • Anticancer • Nephroprotective • Antipyretic • Anti-inflammatory • Anagesic	[83, 84, 85, 86, 87, 88, 89, 90, 91, 92, 93]
*Hygrophila auriculata*	Acanthaceae	Kulekaanta	• Aphrodisiac • Diseases of the urinogenital tract • Dropsy from chronic Bright's disease • Hyperdipsia • Flatulence • Diarrhea • Dysentery • Leukorrhea • Gonorrhea • Asthma • Blood diseases • Gastric diseases • Inflammation • Cancer • Rheumatism • Painful micturition • Menorrhagia	• Antitumor activity • Anti-inflammatory • Antipyretic • Hematopoetic • Hepatoprotective • Diuretic • Antidiabetic • anthelmintic • Antibacterial • Antimotility • Antioxidant • Aphrodisiac • Spermatogenic	[94, 95, 96, 97, 98, 99, 100, 101, 102]
*Trianthema portulacastrum*	Aizoaceae	Gadabani	• Pain • Constipation • Stomachic • Bronchitis • Heart diseases of the blood • Anemia • Inflammation • Piles • Ascites • Liver asthma • Jaundice • Amenorrhea • Helminthiasis • Dropsy • Edema • Vermifuge • Rheumatism • Antidote to alcoholic person • Fever • Corneal ulcers • Itching • Dimness of sight	• Antifungal • Analgesic • Antihyperglycemic • Hepatoprotective • Hypolipidemic • Anticarcinogenic • Anthelmintic • Antioxidant	[103, 104, 105, 106, 107, 108, 109, 110, 111, 112, 113]

## 2 Materials and methods

### 2.1 Plant materials

Five green leafy vegetables were selected for the study namely *Hibiscus sabdariffa (F:Malvaceae), Trianthema portulacastrum (F: Azioaceae), Diplazium esculentum (F: Athyriaceae), Heliotropium indicum L. (F: Boraginaceae) and Hygrophila auriculata (F: Acanthaceae)* belonging to five different families (Table 1)**.** Sample of these species were collected from wild in the coastal region of Dumuria upazila of Khulna city, Bangladesh. All the collected plants were identified by National Botanical Herbarium, Dhaka, Bangladesh (Table 1). The inedible portions were removed and washed with distilled water. The samples were shed dried and dried samples were ground to powder by grinding machine and stored in air tight containers. Twenty five grams of powder of each sample were extracted by maceration using ethanol (EtOH) solvent, followed by filtration and evaporation of solvent using rotary evaporator.

### 2.2 Nutrient contents characterization

Evaluation of chemical composition was performed in triplicate to estimate moisture content, pH, lipid, crude fibre, total ash content, protein and carbohydrate. Moisture analysis was performed using crucibles at 105 °C and lipid content was detected by continuous extraction following the methods established by Association of Official Analytical Chemist [11]. The Micro Kjedahl method was utilized for crude protein determination by using a 6.25 correction factor [11]. Total ash content of samples was measured by muffling in a muffle furnace at 550 °C – 600 °C for 6–8 h and total dietary fibre were measured by using sequential enzymatic digestion. Finally, the determination of total carbohydrates was obtained by the difference, using data from moisture, ash, total lipid, protein and fiber contents [12].

### 2.3 Evaluation of minerals

For estimating of total N content colourimetric determination in kjeldahl digests method was followed according to Baethgen and Alley (1989) [13]. 0.1g of each samples was digested adding catalyst mixture (100:10: 1 of K_2_SO_4_:CuSO_4:_Se) and then the diluted digests were mixed with a weakly alkaline mixtute of Na salicylate and Cl sources to ensure a color reaction. The absorbance was measured in a spectrophotometer using a wavelength of 650 nm. Total Nitrogen content was calculated from the given equation using the factors. Determination of Phosphorous was done using the method established by Murphy and Riley (1962) [14]. Flame photometer (PFP7, Jenway LTD, England) was employed to measure Potassium and Sodium content according to Allen (1974) [15].

### 2.4 Determination of antioxidant components (total flavonoid and tannin)

The content of total flavonoid of extracts were determined according to Aluminum trichloride colorimetric method using quercetin as standard [16] and the content of total tannin was evaluated by Folin-Ciocalteu assay with gallic acid as standard followed by Amorim et al., 2008 [17]. The results have been expressed as quercetin equivalent (mg QE/g dry matter) and galliic acid equivalent (mg GAE/g dry matter) for total flavonoid and tannin content respectively.

### 2.5 DPPH radical scavenging assay

Free radical scavenging activity of ethanolic extracts was determined using stable 2, 2-diphenyl-1-picryl hydrazyl (DPPH) radical [18]. Briefly, 2.0 mg of vegetable extracts was mixed with methanol and various concentrations of extract (0.98–500 μg/ml) were added to 5ml of the methanol solution of DPPH (40 μg/ml). The reaction mixture was vortexed vigorously and the tubes were left at room temperature for 30 min, in dark. The absorbance of the mixture was taken at 517nm using ascorbic acid as positive control. Lower absorbance of the reaction mixture means higher free radical scavenging activity. Percentage inhibition activity was calculated by following equation:

$$\%\text{ ​}inhibition\text{ ​}=\text{ ​}\left[1-\left(Abs_{sample}/Abs_{control}\right)\right]\times 100\%$$

Where Abs_sample_ is the absorbance of the sample material and Abs_control_ is the absorbance of the control reaction (DPPH + Methanol).

### 2.6 Superoxide radical scavenging assay

Superoxide radical activity was determined based on the reduction of NBT according to the method Nishikimi, M., *et al* 1972 [19] followed by slight modification. The superoxide radicals generated by non-enzymatic phenazine methosulfate-nicotinamide adenine dinucleotide (PMS/NADH) system, reduce nitro blue tetrazolium (NBT). The reaction mixture contained NBT solution in phosphate buffer (pH 7.4), NADH, PMS solution prepared in phosphate buffer (PH 7.4) and various concentrations of sample extracts. After incubation for 5 min at 25 °C, the absorbance was measured at 560 nm using an appropriate blank solution. Ascorbic acid was used as positive control. Superoxide radical scavenging activity was calculated according to the following equation:

$$\left[\left(A_{o}-A_{1}\right)/Ao\right]\times 100$$

Where A_o_ is the absorbance of the blank and A_1_ is the absorbance in the presence of the sample of extract and standard.

### 2.7 Hydroxyl radical scavenging assay

Hydroxyl radical generated from FeSO_4_, ascorbate and hydrogen peroxide was evaluated as per a standard method with some modification [20]. Ascorbic acid was used as positive control. The reaction mixture contained 1mL 1.5 mM FeSO_4_ solution, 1 mL extract of leafy vegetables or ascorbic acid solution of various concentrations and was added in the solution containing 0.7 mL of 6 Mm H_2_O_2_ and 0.3 mL of Na-Salicylate. The reaction mixture was incubated for 1h at 37 °C. The hydroxylated salicylate complex was measured by taking the absorbance spectrophotometrically at 562 nm. The percentage scavenging effect was calculated as,

$$Scavenging\text{ ​}activity=\left[1-\left({\text{A}}_{1}-A_{2}\right)/A_{0}\right]\times 100\text{,}$$

Where A_0_ is absorbance of the control (without extract) and A_1_ was the absorbance in the presence of the extract, A_2_ was the absorbance without sodium salicylate.

### 2.8 Reducing power assay

The reducing power was determined by monitoring their capacity to reduce Fe^3+^ following the method described by Hazra, 2008 [16]. Each extract of vegetables (0.01–0.1 mg/ml) in distilled water (1 mL) was mixed with 0.2 M phosphate buffer (2.5 ml, pH 6.6) and 1% potassium ferricyanide (2.5 ml) and the mixture was incubated at 50 °C for 20 min in a water bath. After adding 2.5 ml of 10% trichloroacetic acid, the mixture was centrifuged to terminate the reaction. The 2.5 ml of upper layer of the solution was mixed with 2.5 ml distilled water, and 0.5 ml FeCl_3_ solution (0.1%) and the absorbance was measured at 700 nm after 10 min at room temperature against an appropriate blank solution. Ascorbic acid was used as standard. The higher absorbance indicates higher reducing power.

### 2.9 Statistical analysis

Results were expressed as mean ± SD (Standard deviation). All statistical analysis (except free radical scavenging analysis) were performed using one-way ANOVA analysis followed by Bonferroni's post-hoc comparison tests. Analysis was performed in Prism 5.0 (GraphPad software Inc., San Diego, CA). Results were considered as significant when p < 0.001.

## 3 Results and discussion

### 3.1 Nutrient components analysis

The present study depicts the content of moisture, total lipids, crude protein, available carbohydrates, dietary fiber and total ash in five species of leafy vegetables found in southern coastal region of Bangladesh and the proximate component analysis results are demonstrated in Table 3. The moisture content of the collected vegetables was determined on a dry weight (d.w.) basis (g/100g). It was reported that moisture content is one the most important factor affecting directly the nutrient content [21]. It assists in food digestion and absorption. Moisture content can maintain chemical stability and physical properties of food, prolong enzymatic activity and vitamins in food and even minimize lipid oxidation reactions and non-enzymatic browning reactions [22, 23]. Additionally moisture content is significant to maintain freshness and stability of the food [24]. The moisture content varied from 10.46% for *H. sabdariffa* to 6.20% for *T. portulacastrum.* This moisture value of collected *H. indicum* (8.37%) corroborates with the result which showed that the moisture content was found (8.85%) for West African species *H. indicum* [25]. The present study showed that the lipid content for the collected vegetables was very low and these results confirmed the finding which showed that leafy vegetables are the minor sources of lipids [26]. The lipid content ranged from 5.26% for *H. sabdariffa* to 1.9% for *H indicum* (Table 3)*.* The values of 1.9% and 2.16% for *H. indicum* and *D. esculentum* were similar to 0.675% and 3.40% reported for African *H. indicum* and *D. esculentum* from Philipines [25, 27], whereas the lipid content (5.26%) for *H. sabdariffa* was higher compared to 1.1 % reported for Indian species of *H. sabdariffa* [28]. It is reported that 1–2 % of caloric energy from daily diet as fat is sufficient to human beings because excess fat consumption causes different cardiac diseases and cancer [29]. So the consumption of these green leafy vegetables in huge amount can be recommended to individuals who suffering from diseases such as cardiovascular disorders such as atherosclerosis, cancer and obesity. Leafy vegetables are reported as an indisputably source of protein as leaves synthesis proteins actively and also crude fibre, vitamins and mineral salts [30, 31, 32, 33]. In this study the content of crude protein of the collected five leafy vegetables ranged between 8.01 to 9.44 g/100 g on dry weight basis (Table 3). The value is supported by the findings of protein content for fresh Indian *Hibiscus sabdariffa* and dried Phillipines species of *Diplazium esculentum* were 3.2% and 10.67%, respectively [27, 28]. According to Ali, 2009 plant foods providing around 12% caloric value from protein are considered as a good protein source [34]. Implication of this study is that these measured levels of protein demonstrate the investigated coastal vegetables as alternative sources for cheap and abundant dietary proteins for people of coastal region in Bangladesh. Furthermore if protein is absorbed completely then 100 g of the examined vegetables would contribute fairly 11% of the recommended dietary allowance of protein (71 g/day) for both pregnant and lactating mothers [35]. Dietary fibre level obtained in the analysis was higher in *T. portulacastrum* (24.06%) than in other vegetables such as *H. indicum* (19.98%), *H. sabdariffa* (18.75%), *H. auriculata* (17.36%) and *D. esculentum* (15.59%). The values were higher than the content of fibre reported in West African, Indian or Phillipines species [25, 27, 28]. High levels of crude fibre in diet aid in digestion, bowel problem, constipation and colon cancer [36, 37]. Adequate intake of fibre is advantageous to reduce the risk of heart disease, diabetes, different types of cancer as well to maintain lower cholesterol level [38, 39]. Studies showed repeatedly that diet including low vegetables associates with uprising stomach and colon cancer [40]. In this study it was observed that investigated samples are full of crude fibre therefore leafy vegetables would be recommended for their active role to prevent cardiovascular disease, obesity, diabetes and cancer. According to Khan, et al., 2013 [41] carbohydrates are primary and available source of energy since 4 kCal energy is produced by 1 g of carbohydrates. From the result it was observed that leafy vegetables are highly valued as a good source of carbohydrate. In this study the carbohydrate contents were 59.62%, 58.24%, 53.15%, 50.76% and 46.96% for *D. esculentum*, *H. sabdariffa, H. indicum, H. auriculata* and *T. portulacastrum* respectively. With regards to the recommended dietary allowance for carbohydrate (130 g) according to FAO (1998) [42], these vegetables could be great sources for carbohydrate and could contribute to improve human diet substantially. From biochemical standpoint, ash content is also essential since it is an evidence of minerals [43]. Ash content of collected coastal vegetables were found 5.096%, 6.75%, 7.29%, 10.60% and 11.11% for *D. esculentum, H. indicum, H. sabdariffa, H. auriculata* and *T. portulacastrum* respectively. The values were lower compared to 15.71% and 17.39% in African species *H. indicum* and Phillipine species *D. esculentum* reported in previous investigations [25, 27], but higher than Indian *H. sabdariffa,* and *T. portulacastrum* species [28, 44].

**Table 3:** Comparison of moisture (%) and nutritional proximate content (g/100 g) of collected vegetables from southern coastal region of Bangladesh.

Sample name	Moisture	Lipid	Protein	Ash	Total carbohydrate	Fibre
*H. indicum*	8.37 ± 0.84	2.9 ± 0.38^†^	8.85 ± 0.02*	6.75 ± 0.56*	53.15 ± 0.3^§^	19.98 ± 0.23*
*T. portulacastrum*	6.20 ± 0.64*	3.66 ± 0.34	8.01 ± 0.14	11.11 ± 0.33^†^	46.96 ± 0.3	24.06 ± 0.36^#^
*D. esculentum*	8.8 ± 0.41	2.16 ± 0.43^†^	8.73 ± 0.03*	5.09 ± 0.01*	59.62 ± 0.2*	15.59 ± 0.39*
*H. sabdariffa*	10.46 ± 0.84	5.26 ± 0.34	9.44 ± 0.05*	7.29 ± 1.25	58.24 ± 0.5*	18.75 ± 0.19
*H. auriculata*	9.91 ± 0.44	3.33 ± 0.39	8.01 ± 0.03	10.60 ± 0.39	50.76 ± 0.26	17.36 ± 0.24

Values are mean ± SD. *p < 0.001 vs. *H. auriculata*, ^†^p < 0.001 vs. *H. sabdariffa,*^#^p < 0.001 vs. *D. esculentum,*^§^p < 0.001 vs. *T. portulacastrum.* Data was analyzed by one way ANOVA followed by Bonferroni's test.

### 3.2 Evaluation of minerals

Mineral profile of selected leafy vegetables is presented in Table 4. From the evaluation presence of essential minerals such as N, P, K and Na were confirmed. It is reported that sodium and potassium are the main cations which are located in every cell of the body to regulate the acid-base balance, nerve and muscle contraction and to maintain the plasma volume [45]. Phosphorus is one of the five major minerals in the human body [46]. Phosphorus is the principal element in the structure of the nucleus and cytoplasm of all tissue cells. It serves as a constituent of bones, teeth, adenosine triphosphate (ATP) and nucleic acids. Phosphorus deficiency may cause bone diseases such as rickets in children and osteomalacia in adults. In this study, all the collected coastal vegetables have contained moderate amount of Na and was ranged from 21.52 to 19.976 mg/g for *H. auriculata* and *H. sabdariffa* respectively (Table 4). Therefore, utilization of these leafy vegetables can lessen the possibility of Na deficiency. The highest amount of K was found in *H. auriculata* (13.5 mg/g). *T. portulacastrum, D. esculentum* and *H. sabdariffa* have shown less amount of K than *H. indicum* (10.06 mg/g). The analyzed leafy coastal vegetables have high nitrogen content and the range was 15.116 to 12.82 mg/g. It was revealed that *T. portulacastrum* and *H. auriculata* have the approximately same content of nitrogen (12.82 mg/g). All the collected samples showed very low amount of phosphorus (1.04 – 1.8 mg/g).

**Table 4:** Comparison of mineral content (mg/g) of collected vegetables from southern coastal region of Bangladesh.

Sample name	Nitrogen (N)	Phosphorus (P)	Potassium (K)	Sodium (Na)
*T. portulacastrum*	12.82 ± 0.14^#^	1.78 ± 0.09*	9.6 ± 0.09^#^	21.40 ± 0.20^†^
*D. esculentum*	13.97 ± 0.04*	1.58 ± 0.01*	7.93 ± 0.08^†^	20.21 ± 0.21
*H. sabdariffa*	15.116 ± 0.05*	1.53 ± 0.03*	6.21 ± 0.08	19.97 ± 0.02*
*H. auriculata*	12.82 ± 0.03	1.04 ± 0.03	13.55 ± 0.06^§^	21.52 ± 0.34
*H. indicum*	14.56 ± 0.02^§^	1.8 ± 0.02^†^	10.06 ± 0.48^†^	20.21 ± 0.01^§^

Values are mean ± SD. *p < 0.001 vs. *H. auriculata*, ^†^p < 0.001 vs. *H. sabdariffa,*^#^p < 0.001 vs. *D. esculentum,*^§^p < 0.001 vs. *T. portulacastrum.* Data was analyzed by one way ANOVA followed by Bonferroni's test.

### 3.3 Determination of antioxidant components (total phenol, flavonoid and tannin)

Antioxidants have become very important with regards good health. They are a class of compounds which prevent different types of chemical damage caused by an excess of free radicals. By destroying free radicals, antioxidants may help to prevent cancer, heart disease, stroke and other immune diseases [47]. This is the first time these leafy vegetables in Bangladesh are being investigated of for their phenolic flavonoid and tannin content determination and the results are demonstrated in Table 5.

**Table 5:** Comparison between total flavonoid (mg QE/g dry extract) and tannin (mg GAE/g dry extract) content in the collected vegetable from southern coastal region in Bangladesh.

Sample	Total Flavonoid	Total Tannin
*T. portulacastrum*	144.56 ± 2.21*^,†^	26.34 ± 0.44*
*D. esculentum*	117.91 ± 7.98	44.32 ± 1.16^†^
*H. sabdariffa*	109.52 ± 6.52	57.24 ± 1.16*
*H. auriculata*	128.43 ± 5.10	36.72 ± 0.43
*H. indicum*	90.64 ± 3.75	29.63 ± 1.52

Values are mean ± SD. *p < 0.001 vs. *H. auriculata*, ^†^p < 0.001 vs. *H. sabdariffa.* Data was analyzed by one way ANOVA followed by Bonferroni's test.

Flavonoids are considering as one of the major group of natural phenolics that have been reported to have highest antioxidant activity [48, 49]. In this investigation total flavonoid content of vegetables extract was in ranges between 90.64 -144.56 mg QE/g dry extract (Table 5). *T. portulacastrum* possess high flavonoid content 144.56 mg QE/g dry extract and *H. indicum have* low flavonoid content 90.64 mg QE/g dry extract. Whereas Indian of *H. indicum* extract showed 53.37 mg QE/g and *T. portulacastrum does* not possesses any flavonoids [50]. Localization of flavonoids within the artificial and biological membranes and interaction of flavonoid at the surface of bilayers can act to reduce oxidant by altering cell membrane structures [51]. Thus, flavonoids protect the structure and function of membrane from oxidants.

The tannins, a class of phenolic compounds, are important secondary metabolites with potential bioactivities. They are widely distributed in almost all plant foods, species. Tannins are reported as potential antioxidants and they may reduce the risk of cancer and cardiovascular diseases [52]. Total tannin content of vegetables was found between 26.89 mg/g - 57.29 mg GAE/g dry extract (Table 5). In this study it was investigated that *H. sabdariffa* depicts high 57.29 mg GAE/g dry extract and *T. portulacastrum* have low tannin content 26.89 mg GAE/g dry extract. This is the first time report on total tannin content of these Bangladeshi coastal green leafy vegetables species that content tannin to a different degree.

### 3.4 DPPH radical scavenging assay

Consumption of foods containing antioxidant phytoconstituents is beneficial to human health since they can protect the human body from detrimental free radicals and inhibit the progress of many chronic diseases [53]. The free radical scavenging potential of the collected vegetables was determined using DPPH radical. The DPPH radical is widely used to evaluate the free-radical scavenging capacity of antioxidants according to their hydrogen-donating ability [54]. In addition to that, reactions involved in this method are fully unaffected by side reactions [55]. In this study, it was investigated that the IC_50_ values for the test samples lie in the range between 53.02 and 1561.71 μg/ml (Table 6). Amongst the samples, *T. portulacastrum* showed good free radical scavenging capacity (IC_50_ 53.02 μg/ml) that is near to standard ascorbic acid (IC_50_ 40.16 μg/ml) which indicates that it has potent antioxidant property. This is the first report related to free radical scavenging capacity of these vegetables extracts cultivated in Bangladesh. However, DPPH value of *T. portulacastrum* cultivated in Pakistan showed IC_50_ in the range 6.98 – 311.61 μg/ml [56], *H. auriculata* cultivated in India showed IC_50_ 667.64 μg/ml [57], *D. esculentum* cultivated in India showed IC_50_ 94.94 μg/ml and *H. indicum* cultivated in India showed IC_50_ 30.94 μg/ml [50]. The deviation in results can be due to the difference in extraction technique and assay methods.

**Table 6:** Comparison of IC_50_ (μg/ml) between free radical, superoxide, hydrogen peroxide, reducing power of the leafy vegetable extract collected from southern coastal region in Bangladesh.

Sample name	DPPH free radical scavenging	Superoxide scavenging	Hydroxyl radical scavenging	Reducing power
*T. portulacastrum*	53.02	94.76	42.43	51.7
*D. esculentum*	146.51	111.17	43.45	76.36
*H. sabdariffa*	186.47	118.11	104.88	103.17
*H. auriculata*	156.71	115.48	54.63	69.66
*H. indicum*	37.19	83.44	47.65	145.52

### 3.5 Superoxide radical scavenging assay

Superoxide is a biologically important entity as it can be decomposed to stronger oxidative species such as singlet oxygen and hydroxyl radicals and is very harmful to the cellular components in a biological system [58]. Superoxide anion radical (O^2•-^) develops through reduction of one-electron from free molecular oxygen by a membrane-bound enzyme namely nicotinamide adenine dinucleotide phosphate oxidase. In a cell, it converts to other harmful reactive oxygen species like hydroxyl radical and hydrogen peroxide [59]. Thus, elimination of superoxide radical anion generated by this enzymatic pathway would be beneficial in the case of different health issues. In this study superoxide scavenging capacity i.e. IC_50_ values range between 83.44 to 118.11 μg/ml (Table 6). Results demonstrated that the superoxide scavenging activity of *H. indicum*, *T. portulacastrum*, *D. esculentum*, *H. sabdariffa* and *H. auriculata* increased markedly with the increase of concentrations. Among the samples, IC_50_ values of methanolic extracts of *H. indicum* and *D. esculentum* cultivated in India was reported to be 42 μg/ml and 90.39 μg/ml respectively [60, 61]. This is the first time that these Bangladeshi grown vegetables are evaluated for superoxide scavenging potential.

### 3.6 Hydroxyl radical scavenging assay

Hydroxyl radical scavenging activity of the extract of collected leafy vegetables was evaluated for inhibition of hydroxyl radical generated from FeSO_4_ and hydrogen peroxide systems. The results of hydroxyl radical scavenging activity of all the extracts are shown in Table 6. The figure clearly showed that hydroxyl radicals were scavenged by the extracts in a concentration dependent manner. It can be noted that at lower concentrations, the plant extract possesses higher H_2_O_2_ scavenging activity. The SC_50_ value of hydroxyl radical scavenging activity of vegetable extracts was found highest for *H. sabdariffa* (104.88 μg/ml) while lowest for *T. portulacastrum* (42.43 μg/ml). Whereas SC_50_ value of ascorbic acid was 40.16 μg/ml. *H. indicum* showed SC_50_ 47.65 μg/ml in hydroxyl radical scavenging activity which is much higher than Indian cultivated *Heliotropium indicum* (SC_50_ 23.5 %) [62].

### 3.7 Reducing power activity

The measurements of the reducing ability through Fe^3+^–Fe^2+^ transformation were investigated with the collected samples, *H. indicum*, *T. portulacastrum*, *D. esculentum*, *H. sabdariffa and H. auriculata*. The reducing capacity of a compound may serve as a significant indicator of its potential antioxidant activity. However, the activity of antioxidants has been assigned to various mechanisms such as prevention of chain initiation, binding of transition-metal ion catalysts, decomposition of peroxides, prevention of continued hydrogen abstraction, reductive capacity and radical scavenging [63].

The results demonstrated that the reducing power of vegetables extracts increased with increasing concentration (Table 6). The result obtained from the experiment indicates that the vegetable extracts have some reducing capacity with RC_50_ ranging between 51.7- 145.2 μg/ml. In this investigation *T. portulacastrum* (51.7 μg/ml) showed the most potent reducing capacity.

## 4 Conclusion

In the present nutritional proximate analysis, the results demonstrated that out of the five coastal species studied, *T. portulacastrum* recorded as higher amount of ash and fibre content, while *D. esculentum and H. sabdariffa* recorded as higher amount of protein and carbohydrate content. In the mineral content *H. auriculata* showed to possess highest amount of potassium and sodium whereas, *T. portulacastrum* showed higher amount of phosphorus and sodium content. Interestingly, all the investigated vegetable species showed low amount of lipid content.

A number of antioxidant experimental model were assessed for the first time to evaluate the antioxidant potential of these plant extracts. With the results obtained, it can be concluded that out of the five plants, *T. portulacastrum* showed significant scavenging activity in superoxide radical, reducing power and hydrogen peroxide scavenging assays. It also recorded higher amount of flavonoids content which might be contributed to its free radical scavenging or reducing power activity. However, other plant species also showed comparable antioxidant potential in terms of free radical scavenging and antioxidant components content to the reported some of these species cultivated around the world. The results we obtained in this study clearly indicate that common plants used as vegetable by the people of southern coastal region of Bangladesh are rich in protein, fibre and carbohydrate as well as a good source of antioxidant components. However, there could be a variation of these proximate content and antioxidant capacity (quality) of these coastal vegetables with other sources because of environment, salinity, cultivated medium, climate of cultivation country, as well as the preparation procedure. Therefore, these plants can be promoted as a significant source of nutritional and antioxidant food supplements in the coastal region of Bangladesh.

## Declarations

### Author contribution statement

S.M. Neamul Kabir Zihad: Performed the experiments; Analyzed and interpreted the data; Wrote the paper.

Yashu Gupt, Md. Rabiul Alam, Mahmood Hossain: Performed the experiments; Analyzed and interpreted the data.

Shaikh Jamal Uddin: Conceived and designed the experiments; Contributed reagents, materials, analysis tools or data; Wrote the paper.

Muhammad Torequl Islam: Analyzed and interpreted the data; Wrote the paper.

Shahin Aziz: Analyzed and interpreted the data.

Jamil A. Shilpi, Lutfun Nahar, Satyajit D. Sarker: Conceived and designed the experiments; Contributed reagents, materials, analysis tools or data.

### Funding statement

This research did not receive any specific grant from funding agencies in the public, commercial, or not-for-profit sectors.

### Competing interest statement

The authors declare no conflict of interest.

### Additional information

No additional information is available for this paper.
